# Draft Genome Sequence of Legionella pneumophila D-5864, a Serogroup 6 Strain

**DOI:** 10.1128/genomeA.01379-14

**Published:** 2015-01-08

**Authors:** Shatavia S. Morrison, Natalia A. Kozak-Muiznieks, Scott Sammons, Lori A. Rowe, Mike Frace, Jonas M. Winchell

**Affiliations:** a Division of Bacterial Diseases, National Center for Immunization and Respiratory Diseases, Centers for Disease Control and Prevention, Atlanta, Georgia, USA; b Biotechnology Core Facility Branch, Division of Scientific Resources, National Center for Emerging and Zoonotic Infectious Diseases, Centers for Disease Control and Prevention, Atlanta, Georgia, USA

## Abstract

Legionella pneumophila is the leading etiology of legionellosis infections in North America and Europe. Here we report the draft genome sequence of L. pneumophila D-5864, a serogroup 6 strain, which was isolated from a bronchial alveolar lavage specimen of a male patient from Arizona in 2009. Genes within the lipopolysaccharide (LPS)-biosynthesis region could potentially be determinants of serogroup specificity.

## GENOME ANNOUNCEMENT

Legionella pneumophila is a Gram-negative bacterium found in natural freshwater environments worldwide. However, it can also colonize manmade water systems such as cooling towers, whirlpool spas and potable water systems (1). In man-made water systems, L. pneumophila can be transmitted into a susceptible human host by inhalation of aerosolized water containing this organism (1). Several studies have shown that L. pneumophila serogroup (sg) 6 is the second most common serogroup associated with clinical cases, following only the well-studied and documented sg 1 (2–6). Here we report a draft genome sequence of L. pneumophila strain D-5864 (sg6). This strain was isolated from the lower respiratory tract (bronchial alveolar lavage) of a male patient in Arizona in 2009. This draft genome will be helpful to the Legionella research community to aid in research efforts to elucidate functional and pathogenic differences within this serogroup and allow a more comprehensive understanding of the genetics of different legionellae, including other serogroups of L. pneumophila. Further analyses of these data may also lead to improvements in the characterization and detection methods for all L. pneumophila serogroups.

The genome was sequenced using the Roche 454-pyrosequencing (Branford, CT, USA) and Illumina (San Diego, CA, USA) sequencing platforms. After removal of low-quality sequence reads and read trimming, a total of 12,086,080 single-end reads were used for the downstream analysis. A *de novo* assembly was performed using CLC Genomics which resulted in a draft genome of 35 contigs, with a total length of 3,390,488 bp and a G+C content of 38.2%. The genome sequence was annotated by the Rapid Annotations using Subsystem Technology (RAST) server (7). RAST identified 3,292 genomic features consisting of 3,249 coding sequences (predicted), 40 tRNAs, and 3 rRNAs. Also, we mapped sequence reads to the genome of L. pneumophila Thunder Bay sg 6 (8) to show relatedness. The mapping resulted in a high sequence similarity of approximately 98.8%.

The LPS-biosynthesis region has been shown to be useful in the classification of L. pneumophila into serogroups and subgroups by monoclonal antibodies (9–11). A correlation between the presence and absence of genes within the LPS biosynthesis region may be linked to L. pneumophila serogroup classification (12). In a gene comparison of this region between L. pneumophila strains Philadelphia (sg1), Paris (sg1), Thunder Bay (sg6), and D-5864 (sg6), we identified a similar pattern of gene presence/absence as seen by Cazalet et al. (12). While both sg 1 strains contained all 28 genes of the LPS region (*lpp*0814–*lpp*0843 as per L. pneumophila Paris nomenclature), 13 out of 28 genes were present in both sg 6 strains (*lpp*0814–*lpp*0826). An in-depth comparative genomics study will be performed in the future.

### Nucleotide sequence accession numbers.

This whole-genome shotgun project has been deposited at GenBank under the accession number JRMJ00000000. The version described in this paper is version JRMJ01000000.
